# Improving Vitamin D Screening in a Pediatric Rheumatology Clinic Using Structured Quality Improvement Process

**DOI:** 10.1097/pq9.0000000000000594

**Published:** 2022-09-08

**Authors:** Aliese Sarkissian, Edward Oberle, Ohoud Al Ahmed, Dawn Piccinich, Fatima Barbar-Smiley, Helen Zak, Vidya Sivaraman

**Affiliations:** From the * Division of Rheumatology, University of North Carolina at Chapel Hill, Chapel Hill, North Carolina; † Nationwide Children’s Hospital and The Ohio State University Medical Center, Columbus, Ohio; ‡ Catalysis, Appleton, Wis.

## Abstract

**Introduction:**

Monitoring levels of 25-hydroxyvitamin D (25-OHD) is an integral part of bone health assessment in the general pediatric population, especially in at-risk populations such as children with juvenile idiopathic arthritis (JIA), childhood-onset systemic lupus erythematosus (c-SLE), and juvenile dermatomyositis (JDM). However, only 38% of the patients with JIA, c-SLE, and JDM receiving care at Nationwide Children’s Hospital Rheumatology clinic in 2016 had a 25-OHD level ordered in the preceding year. The objective of this project was to increase the percentage of 25-OHD levels ordered in patients with JIA, c-SLE, and JDM from 38% to 80% in 11 months and sustain it for 6 months.

**Methods:**

A multidisciplinary team initiated a continuous improvement project utilizing the Lean Six Sigma methodology. The team diagrammed the clinical process and identified steps that needed improvement. In addition, the team completed a root cause analysis of the process and brainstormed subsequent countermeasures.

**Results:**

The team did not meet the 80% target but did order a 25-OHD level on 61% of patients by the end of the study period compared to 38% at the start of the study (*P* value 0.001). The level was sustained after the study period, with 68% of these children having a 25-OHD level ordered.

**Conclusion:**

The team successfully improved the screening processes for vitamin D deficiency in a busy subspecialty clinic setting using Lean Six Sigma methodology.

## INTRODUCTION

Studies show vitamin D insufficiency in children and adolescents is a health problem worldwide, with 50% of children ages 1–5 years and 70% of children ages 6–11 years having a blood level of 25-OHD less than 30 ng/mL.^1^ Vitamin D is required for bone health and may also serve as a modulator in immune responses.^2,3^ Pediatric risk factors for low vitamin D include obesity, decreased sun exposure, and nutritional status.^1–3^

Patients with rheumatologic disorders have unique risk factors for low vitamin D and poor bone health specific to their disease.^1,2,4,5^ Patients with juvenile idiopathic arthritis (JIA), childhood-onset systemic lupus erythematosus (c-SLE), and juvenile dermatomyositis (JDM) may experience limited mobility due to their disease activity. They may be exposed to systemic glucocorticoids to control their disease and are risk factors for poor bone health.^6^ Moreover, ultraviolet light exposure can flare autoimmune diseases; therefore, patients with c-SLE and JDM are frequently counseled about sun avoidance to decrease their risk of disease flare, which in turn limits one major source of vitamin D.^6^ A prospective longitudinal study of 134 pediatric patients with rheumatic disorders demonstrated their risk as an unadjusted vertebral fracture incidence rate of 4.4 per 100 person-years, with a 3-year incidence of 12.4%.^7^

Bone health monitoring in patients with rheumatologic disorders includes recommendations for periodic assessment of their 25-OHD levels ranging from every 6 months to 2 years based on the patient’s other risk factors.^6^ The best method to determine vitamin D status and need for supplementation is by measuring the serum level of 25-OHD.^5^ However, only 38% (1,144/2,983) of the patients with JIA, c-SLE, and JDM seen at a Nationwide Children’s Hospital (NCH) Rheumatology clinic in 2016 had a 25-OHD level ordered in the preceding year. To increase the rate of vitamin D screening, we initiated a continuous improvement project utilizing Lean Six Sigma (LSS) methodology.^8^

LSS combines two methodologies: Lean and Six Sigma, each with complementary strengths that have shown usefulness in healthcare.^8–11^ Lean offers standard solutions to common organizational problems and provides a total system approach to focus on what the customer wants. Six Sigma offers a structured approach to problem-solving deployed in five phases: define, measure, analyze, improve, and control (DMAIC).^8^ Combined, LSS, focuses on processes to reduce errors or variations and add value and efficiency to the workflow.^12^ Our objective was to increase the percentage of JIA, c-SLE, and JDM patients with a 25-OHD level ordered from 38% to 80% in 11 months and sustain this percentage for 6 months. Unfortunately, we could not find a baseline comparison after an exhaustive literature search. Therefore, we set a goal of 80% based on departmental provider consensus. In addition, we chose 11 months based on the necessity of project completion for a concomitant graduate program.

## METHODS

The project included patients diagnosed with JIA, c-SLE, and JDM seen at a rheumatology clinic in a large quaternary academic children’s hospital in Columbus, Ohio. The clinic providers consisted of nine board-certified pediatric rheumatologists, two nurse practitioners, and six rheumatology fellows. The clinic staff included two registration clerks, four nurses, and one medical assistant. Additionally, the hospital’s other registration and nursing staff rotate through the clinic based on daily staffing needs. The clinic saw an average of 208 patients with JIA, c-SLE, or JDM each month during the study period. Baseline analysis included data from January 1, 2016, through December 31, 2016. The study period was from January 1, 2017, to November 30, 2017. The project team leader (Dr. Sarkissian) chose this date as it coincided with completing coursework in LSS. We included data from December 1, 2017, through August 31, 2018, for follow-up analysis.

### Project Team

The hospital has a strong focus on quality and patient safety. Physician fellows are required to attend a 2-hour quality improvement educational session. In addition, attending physicians can participate in quality improvement educational courses. This project was the first time the team members had employed LSS methodology. The project team leader taught the blended methodology to team members before and during monthly meetings while concurrently taking classes in LSS. By the end of the study period, the project leader was Black Belt certified in LSS. Project team members included three attending physicians (one of whom completed the hospital’s 5-month QI certificate program), two rheumatology fellows, and one clinic nurse.

### Problem Solving

**Fig. 1.**
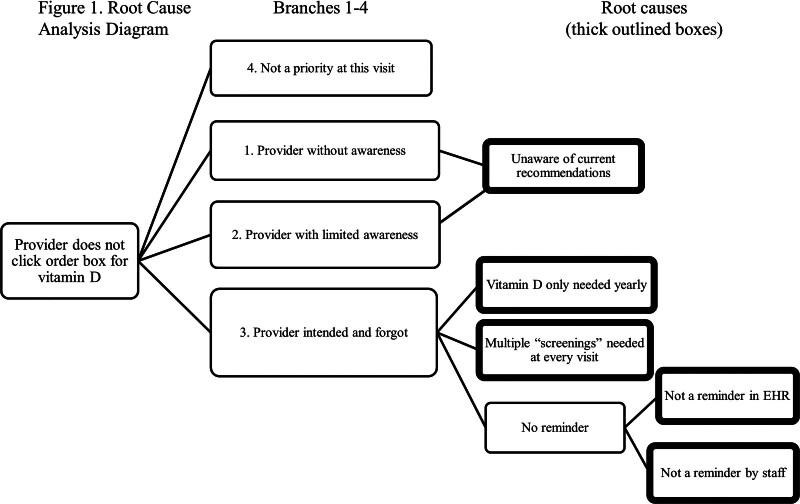
Root cause analysis diagram.

**Fig. 2.**
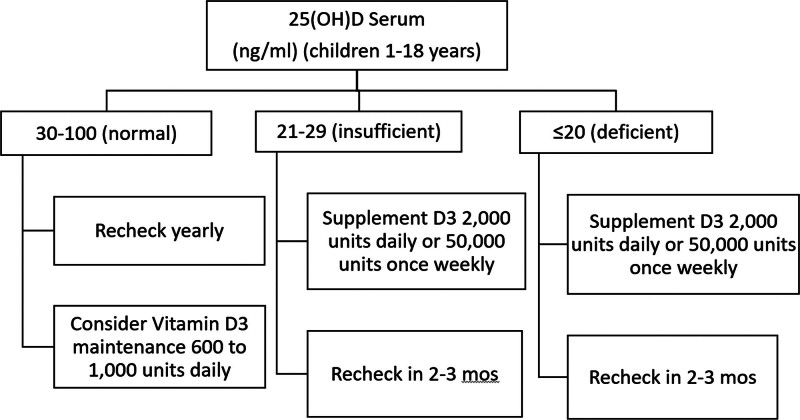
Vitamin D monitoring algorithm.

The project team utilized an A3 template,^13^ a one-page summary report used in LSS (see figure 1, Supplemental Digital Content 1, http://links.lww.com/PQ9/A402, which describes A3), to problem solve, organize, and communicate project progress. Value stream mapping helped the team visualize the process from when a patient arrives in the clinic to when the patient receives their laboratory results after their visit (see figure 2, Supplemental Digital Content 2, http://links.lww.com/PQ9/A403, which describes value stream map). The mapping process identified multiple bottlenecks throughout the process. The overall process time, which includes the process steps, such as when the patient is seen, and laboratories are ordered, was very low compared to the production time, or the total time to complete the process steps, due to long wait times between each process step. Our team focused efforts on a bottleneck at the beginning of the value stream, when a provider orders laboratories for a patient, since this prevented further process steps from occurring. As a result, the team noted only 38% of patients had a vitamin D level ordered by their provider at their visit.

To ensure that the countermeasures targeted the sources of the bottleneck, the team performed a root cause analysis (RCA) for the process step “provider does not check order box for vitamin D.” The team conducted a group brainstorming session with follow-up staff interviews to complete the RCA. The original RCA included many branches and, for simplicity, is distilled into the RCA (Fig. 1). The RCA yielded four main branches: (1) the provider was unaware of the need for screening in our population; (2) the provider was aware of screening but, through prior experience, did not think it was necessary; (3) the provider was fully aware and intended to order it but forgot; and (4) the provider was fully aware of the screening need and intended to order it, but other visit aspects (eg, disease management) took priority. The team did not include the fourth branch because it was beyond the project’s scope.

To address the root cause of branches (1) and (2) that providers were unaware of current recommendations, the team brainstormed countermeasures to educate staff about the importance of vitamin D screening and supplementation in this population. These educational measures included an informational e-mail about vitamin D screening and a presentation at a divisional meeting, including all rheumatology department staff. The educational component represented the first countermeasure.

**Fig. 3.**
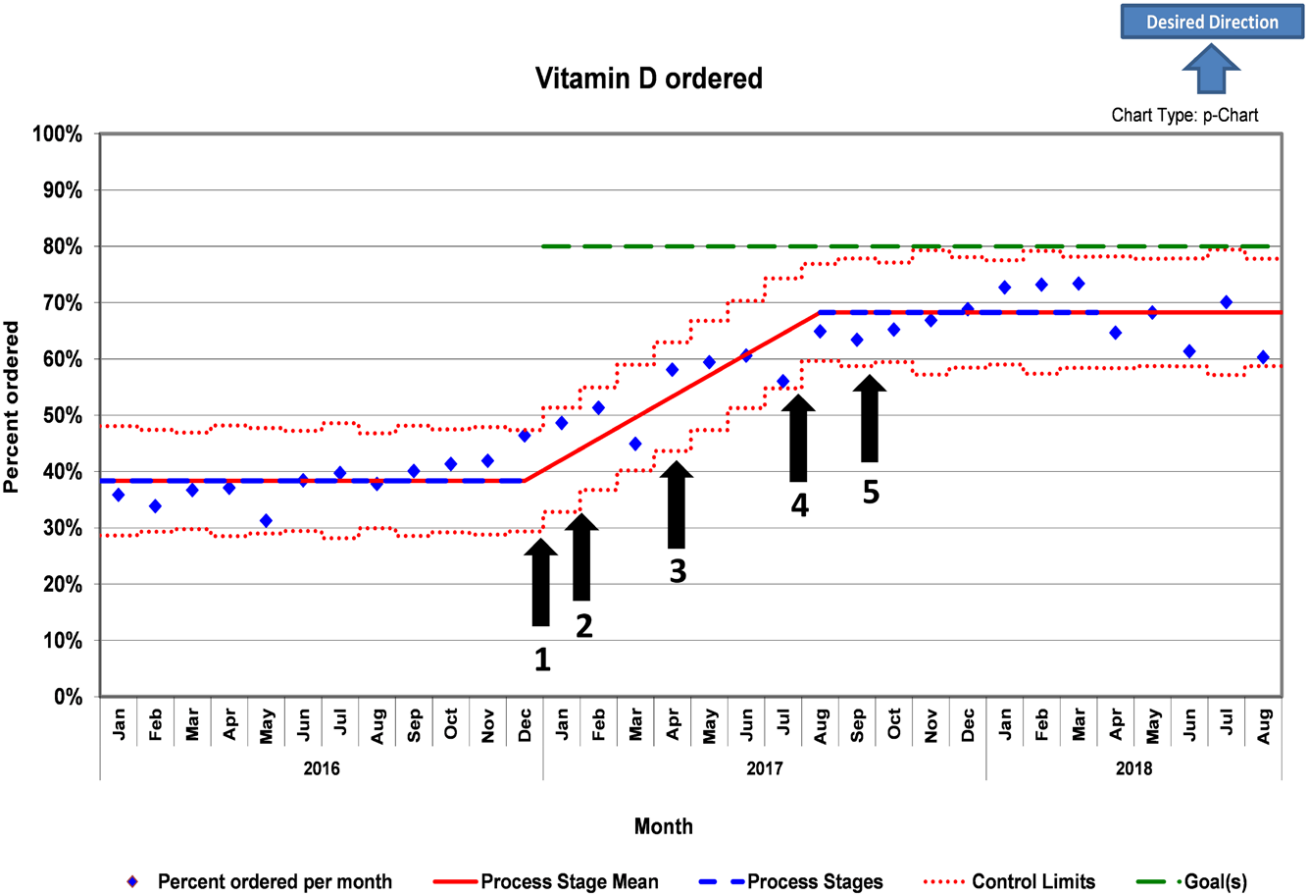
p-Chart of percentage of patients with vitamin D ordered; 1. Project conception; 2. Team formed; 3. Visual reminders placed and intake sheet created; 4. Algorithm posted in clinic; and 5. EHR phrase created.

To address branch (3) root cause, in which the provider is fully aware of the need and forgets despite best intentions, the team initially focused on three countermeasures (the second, third, and fourth overall countermeasures). For the second countermeasure, the team installed a visual prompt on clinic computer workstations that asked providers, “Have you checked my Vitamin D?” Next, for the third countermeasure, they developed a screening and treatment algorithm for vitamin D screening and supplementation (Fig. 2). Last, the fourth countermeasure utilized the clinic nurse’s feedback to construct a standard nursing intake sheet to address the provider task burden. This tool was used for all patients regardless of diagnosis and included a space for the patient’s last vitamin D level (see figure 3, Supplemental Digital Content 3, http://links.lww.com/PQ9/A404, which describes nursing intake sheet). The team instructed the nursing staff to determine when vitamin D levels were last checked and include the result during their intake process.

In the previous process, the nurses wrote notes for the providers about the patient on a demographic sheet when a patient checked in. The new intake sheet included other quality measures that the hospital and the department were tracking, such as when certain vaccines were due. Clinic nurses took ownership of the intake sheet and updated it as clinic needs changed. In addition, the team trained nurses to search the patient’s electronic health record (EHR) for the patient’s vitamin D level and record it on the sheet. Because of the intake sheet and the algorithm, providers could easily review the level and when it was last drawn. This information allowed them to decide if it was time for a recheck and whether the patient needed supplementation. It also provided an additional reminder to check the patient’s vitamin D level. Ultimately, the goal was to shift the intake sheet to the EHR. However, this change was not possible in the period for project completion.

Alternatively, using a smart phrase as a countermeasure was feasible within the project period and was the fifth countermeasure, addressing root cause branches (1) and (2). The team drafted a smart phrase with easy readability for patients and families, which included education about vitamin D and the patient’s last level checked. The team distributed the smart phrase to all providers for consensus. The smart phrase was made available for all providers to use to check their patient’s last vitamin D easily and to provide education to patients and their families. The final smart phrase was as follows:

Vitamin D helps the bones stay strong and healthy. Children with rheumatic diseases can be at risk for thinning of the bones (osteopenia). This occurs due to their disease, medications such as steroids, and decreased sun exposure or exercise. We will measure your child’s Vitamin D level with their normal blood work. A level above 30 ng/mL is normal for children 1 to 18 years old. Your child can also build strong bones by eating foods rich in calcium and vitamin D and getting 30-60 minutes of exercise every day.

Your most recent level: @LASTLAB(vitd25hydrox:1)@

### Data Collection

Providers usually screen patients with normal vitamin D levels annually, whereas other patients may require more frequent monitoring, such as after receiving vitamin D supplementation. This variation in monitoring frequency challenged measuring countermeasure success in real-time. Therefore, the team collaborated with hospital information services to extract EHR data, including clinic visit date and if a provider ordered 25-OHD at that visit or 1 year prior for patients with JIA, c-SLE, and JDM. The team identified patients based on billing diagnosis codes entered by the provider. The team included the year before allowing new patient visits to be added while not removing ones already screened. The Information Services department released monthly reports with a 2-week delay to ensure that billing data correctly reflected patients with the desired diagnoses. The team defined patients as “screened” if they had a 25-OHD ordered at or within 1 year of their clinic visit. Finally, the team compared “screened” patients with the total number of patients with JIA, c-SLE, and JDM seen in the clinic over 1 month.

The team used a statistical process control p-chart utilizing NCH proprietary software with trend detection to track patients screened at visits. The chart represents the percentage of patients where Vitamin D is ordered starting March of 2021 with a baseline of one year from January 2016 to December 2016 (Fig. 3). A stable data period before and after the first intervention established the initial baseline. A shift started in August 2017 after a pattern of eight consecutive points was detected with a similar result. A two proportions test was used to calculate the *P* value of the center line shift.

The project was performed for quality improvement and was not considered human subject research. Therefore, per NCH’s institutional policy, it was considered exempt from Institutional Review Board approval.

## RESULTS

### Prestudy Data: 2016

There were 2983 visits with patients diagnosed with JIA, c-SLE, and JDM in the rheumatology clinic in 2016. However, baseline data from 2016 showed that only 1134 (38%) visits with patients with JIA, c-SLE, and JDM had a vitamin D checked in the preceding year.

### Poststudy Period: December 2017 to August 2018

Although the study ended on November 30, 2017, monthly data collected from December 2017 through August 2018 demonstrated significant change and sustained improvement, staying above 60%, with 1,213 of 1,787 (67.8%) patient visits with a 25-OHD level ordered (Fig. 3). In addition, we achieved a statistically significant centerline shift with *P* value (<0.0001) via two proportions test, from approximately 38% baseline to a new baseline of 68% from August 2017 to July 2018. We continue to use the intake forms and update providers about the improvements in screening at division meetings. In addition, further revisions to the intake forms now track the patient’s last eye examination, need for vaccines such as influenza and pneumococcal vaccine, and contraceptive counseling and have even been adapted for telemedicine for other quality improvement projects.

## DISCUSSION

Although measuring 25-OHD levels is integral to assessing bone health in the general pediatric population, 25-OHD monitoring is especially important in at-risk populations such as children with JIA, c-SLE, and JDM. This quality improvement study used LSS methodology to increase the percentage of patients with JIA, c-SLE, and JDM with a 25-OHD level ordered from 38% to 68% in 11 months with sustainment for 6 months. Employment of LSS helped us analyze the process, uncover the problems, and identify root causes that lead to effective countermeasures.

Our main limitations included inexperience with using the LSS methodology, lack of system change countermeasures (changes in EHR/best practice alerts), and project timelines bound by the team leader’s requirements for educational training (completion of the master’s program and graduation of fellowship). In terms of inexperience with LSS methodology, we lacked experience with data measurements. Our outcome measure had a 2-week delay, making it difficult to determine real-time countermeasure success. However, this delay was essential to ensure data accuracy, as billing codes were necessary to verify we were monitoring patients with the appropriate diagnoses. Another measurement limitation to our study is that we did not collect data on the effect the new intake sheet had on time for staff to room patients. It was only subjectively noted through interviews of staff and providers that project implementation maintained clinic flow despite the additional intake form questions. Finally, project timeline restraints led to multiple countermeasures introduced over a short period, which made the interpretation of each countermeasure outcome unclear. Furthermore, without system change countermeasures, sustainment is more at risk of fading over time.

Despite not reaching the team goal of 80% within the 11 months, an increase from 38% to 68% of at-risk patients with a 25-OHD level ordered was a significant and sustainable improvement accomplished by the end of the 6-month follow-up period (May 2018). An assessment of the monthly percentages of patient visits with 25-OHD ordered reflects the success of the countermeasures (Fig. 3). It is important to highlight that multiple countermeasures were introduced in a short time frame, so we cannot necessarily attribute each gain to a specific countermeasure. However, we can compare the success of the countermeasures with the slight improvement after project conception and team formation before any countermeasures. This improvement reflected the raised awareness of the problem, known as the Hawthorne effect.^14^ In contrast, the countermeasures that affected the process and involved more of the care team led to higher gains. For example, developing an intake sheet utilized all team members, distributing the workload, and improving team engagement. The screening and treatment algorithm also reduced process variation and facilitated therapeutic decision-making at the point of care, resulting in improved quality of patient care.

Similarly, in a review of the LSS literature, Deblois and Lepanto^12^ noted successful LSS projects include participation from the entire personnel. Furthermore, the principles of LSS have demonstrated success across an academic hospital in the Netherlands. When the hospital embraced the principles of LSS, they observed improvements such as reductions in the average duration of stay for patients in the wards, wait times for outpatient clinics, surgical treatment for hip fractures, and late operation starts.

Our study also demonstrates the application and usefulness of LSS in the distinctive setting of a busy pediatric specialty clinic. In contrast, most LSS studies have been reported in surgery^15^ and radiology settings.^16^ Furthermore, this study demonstrates countermeasures not used in the previous literature to improve vitamin D screening.^17,18^ Lansdown et al^17^ describe countermeasures utilizing educational reminders and monetary incentives for providers, whereas Stephens et al^18^ describe educational reminders and computerized order sets. Neither study takes advantage of the entire care team’s expertise and distributes tasks among them.

Although LSS methodology was new to our quality improvement toolbox, the observed improvement demonstrated the utility of this methodology in improving care processes. Our team was able to draw parallels with more familiar tools used in Institute for Healthcare Improvement (IHI), such as a key driver diagram (see figure 4, Supplemental Digital Content 4, http://links.lww.com/PQ9/A405, which describes key driver diagram). This study demonstrates that LSS methodology can improve compliance with recommended screenings in a busy pediatric subspecialty clinic.

## DISCLOSURE

The authors have no financial interest to declare in relation to the content of this article.

## Supplementary Material



## References

[1] Holick MF. The vitamin D deficiency pandemic: approaches for diagnosis, treatment and prevention. Rev Endocr Metab Disord. 2017;18:153–165. 28516265 10.1007/s11154-017-9424-1

[2] Antonucci R Locci C Clemente MG. Vitamin D deficiency in childhood: old lessons and current challenges. J Pediatr Endocrinol Metab. 2018;31:247–260. 29397388 10.1515/jpem-2017-0391

[3] Misra M Pacaud D Petryk A; Drug and Therapeutics Committee of the Lawson Wilkins Pediatric Endocrine Society. Vitamin D deficiency in children and its management: review of current knowledge and recommendations. Pediatrics. 2008;122:398–417. 18676559 10.1542/peds.2007-1894

[4] Saraff V Högler W. Endocrinology and adolescence: osteoporosis in children: diagnosis and management. Eur J Endocrinol. 2015;173:R185–R197. 26041077 10.1530/EJE-14-0865

[5] Vojinovic J Cimaz R. Vitamin D—update for the pediatric rheumatologists. Pediatr Rheumatol Online J. 2015;13:18. 26022196 10.1186/s12969-015-0017-9 PMC4446840

[6] Cassidy JT. Textbook of Pediatric Rheumatology. 6th ed. Saunders; 2011.

[7] LeBlanc CM Ma J Taljaard M; Canadian STeroid-Associated Osteoporosis in Pediatric Population (STOPP) Consortium. Incident vertebral fractures and risk factors in the first three years following glucocorticoid initiation among pediatric patients with rheumatic disorders. J Bone Miner Res. 2015;30:1667–1675. 25801315 10.1002/jbmr.2511 PMC4556451

[8] de Koning H Verver JP van den Heuvel J. Lean six sigma in healthcare. J Healthc Qual. 2006;28:4–11. 10.1111/j.1945-1474.2006.tb00596.x 16749293

[9] Tagge EP Thirumoorthi AS Lenart J. Improving operating room efficiency in academic children’s hospital using Lean Six Sigma methodology. J Pediatr Surg. 2017;52:1040–1044. 28389078 10.1016/j.jpedsurg.2017.03.035

[10] Ng D Vail G Thomas S. Applying the Lean principles of the Toyota Production System to reduce wait times in the emergency department. CJEM. 2010;12:50–57. 20078919 10.1017/s1481803500012021

[11] Agarwal S Gallo JJ Parashar A. Impact of lean six sigma process improvement methodology on cardiac catheterization laboratory efficiency. Cardiovasc Revasc Med. 2016;17:95–101. 26905051 10.1016/j.carrev.2015.12.011

[12] Deblois S Lepanto L. Lean and Six Sigma in acute care: a systematic review of reviews. Int J Health Care Qual Assur. 2016;29:192–208. 26959898 10.1108/IJHCQA-05-2014-0058

[13] Shook J. Managing to Learn: Using the A3 Management Process to Solve Problems, Gain Agreement, Mentor and Lead. 1.0. ed. Lean Enterprise Institute; 2008.

[14] Wickström G Bendix T. The “Hawthorne effect”–what did the original Hawthorne studies actually show? Scand J Work Environ Health. 2000;26:363–367. 10994804

[15] Mason SE Nicolay CR Darzi A. The use of Lean and Six Sigma methodologies in surgery: a systematic review. Surgeon. 2015;13:91–100. 25189692 10.1016/j.surge.2014.08.002

[16] Amaratunga T Dobranowski J. Systematic review of the application of Lean and Six Sigma quality improvement methodologies in radiology. J Am Coll Radiol. 2016;13:1088–1095.e7. 27209599 10.1016/j.jacr.2016.02.033

[17] Lansdown DA Whitaker A Wustrack R. A resident-led initiative improves screening and treatment for vitamin D deficiency in patients with hip fractures. Clin Orthop Relat Res. 2017;475:264–270. 27549989 10.1007/s11999-016-5036-4 PMC5174045

[18] Stephens JR Williams C Edwards E. Getting hip to vitamin D: a hospitalist project for improving the assessment and treatment of vitamin D deficiency in elderly patients with hip fracture. J Hosp Med. 2014;9:714–719. 25196298 10.1002/jhm.2255

