# Supplementary material for: Improving Vitamin D Screening in a Pediatric Rheumatology Clinic Using Structured Quality Improvement Process
Source: Pediatr Qual Saf. 2022 Sep 8;7(5):e594. doi: 10.1097/pq9.0000000000000594 (PMC10997281; doi:10.1097/pq9.0000000000000594)
Supplement: Supplementary file 1 [file pqs-7-e594-s001.pdf]

Improving bone health in pediatric rheumatology patients

Owner: Sarkissian   Team: Oberle, Barbar-Smiley, Al-Ahmed, Thomas, Piccinich   Sponsor: Sivaraman   Rev 28, 11/8/2017

Background:

Vertebral fractures are a manifestation of osteoporosis in pediatric patients with chronic inflammatory diseases such as juvenile idiopathic arthritis (JIA), pediatric systemic lupus erythematosus (pSLE), and juvenile dermatomyositis (JDM).[1]

Their specific risk factors	Their general risk factors
inflammation caused by their primary disease<br>glucocorticoids used in their treatment<br>decreased mobility due to their primary disease<br>avoidance of sunlight which can flare their disease	childhood obesity<br>**low vitamin D levels**

-Vitamin D supplementation is recommended as an adjunct to regular inflammatory disease treatment[2]

-One proposed method to address low vitamin D is yearly screening for patients with a normal level and if low, supplementation with a recheck of levels in 3 months to assess response.

-At Nationwide Children's Hospital (NCH), our mission statement is: *"Best outcomes in everything we do, together delivering the best healthcare for children"*

Current Situation:

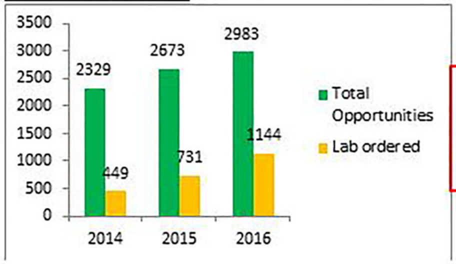

Problem statement: In 2016, only 38% of the total population of JIA, pSLE, and JDM had their vitamin D level screened, a gap of 62% of the total population.

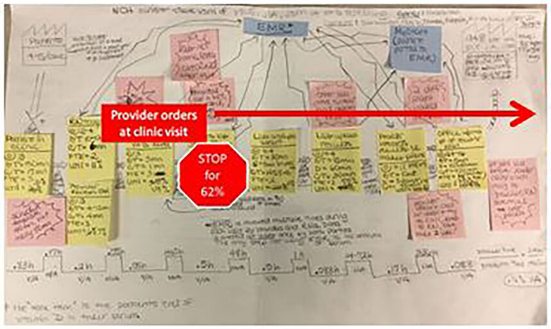

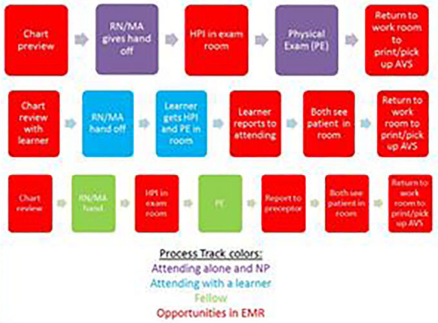

Goal/Target: NCH will screen for Vitamin D levels on 80% of the total population of JIA, pSLE, and JDM by November 1st, 2017.

Analysis:

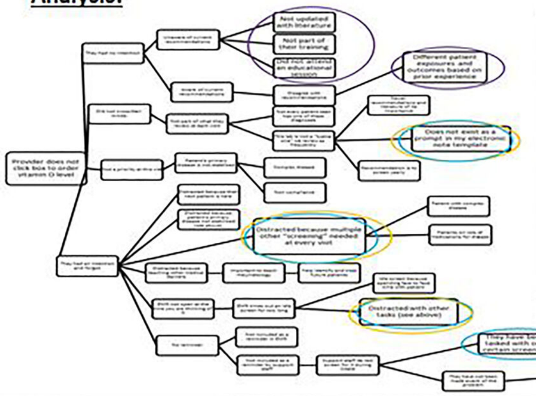

Recommendations:

Counter measures:

-Organize educational session (presentation from "expert") for whole division**
-Updates at division meeting*
-Educational email*
-Educational poster in clinic that patients and families can see**
-Organize journal club of recent literature*
-Report during division meeting (see above)
-Have patients with fractures due to low vitamin D come to division meeting and share experience***
-Report of shared division's experience with patients for whole division***
-Send e-mail with current recommendations and ask providers thoughts to open discussion*
-Have an informational power point slide up at Division meeting **
-Create visual of screening recommendations and treatment algorithm for RNs, providers, staff, and patients**
-Create board of all QI initiative and research***
-Visual reminders on clinic computers*
-Make a "dot phrase" on vitamin D screening to add to notes
-Update our clinic note template to include vitamin D screening ***
-Create standard checklist of monitoring labs/screenings for RNs and providers**
-Create RN standard patient check-in sheet with space for when vitamin D last screened*

Rating of difficulty to complete based on resources and time: (*=easy, **=moderate, ***=hard)

Plan:

Counter measures: Educate, Remind, System	Owner	Assigned	Outcome expected	Began	Status*	Outcome observed	Comments
Visual reminder on clinic room computers	AS	4/13/17	inc 1%	4/20/17		0.065%	put on rest of computers 4/21, 4/24 (when rooms open)
RN standard check-in sheet with screenings included	AS/DP	4/18/17	inc 5%	4/21/17		0.065%	update assigned to DP on 6/9 and completed
Division e-mail education blurb/update	AS	4/13/17	inc 1%	4/24/17		0.060%	sent out algorithm via e-mail
Division meeting update	AS	4/13/17	inc 1%	4/24/17		0.060%	updated on 5/8/17
Assess metric validity	AS/VS	6/8/17	N/A	6/15/17		N/A	updated metrics demonstrated lower baseline
Division meeting education of screening	AS/OA	4/18/17	inc 1%	7/10/17		4.4%	presented work at monthly QI meeting
Have guidelines/algorithm posted in clinic	AS/VS	6/8/17	inc 2%	7/17/17		4.4%	posted!
EPIC dot phrase about screening	AS/VS	6/8/17	inc 2%	8/1/17		1.7%	final one shared on 9/19/17
Presentation to families at "Arthritis Family Day"	AS/VS	5/9/17	inc 0.5%	8/19/17		1.7%	
Send update email on progress	AS	9/28/17	inc 0.5%	9/29/17		0.4%	sent out on 9/29
Update clinic note template with space for screening	AS/EO	4/18/17	inc 5%	9/28/17			discussion started

*Yellow=Horizon Green=ongoing Blue= completed

Follow-up:

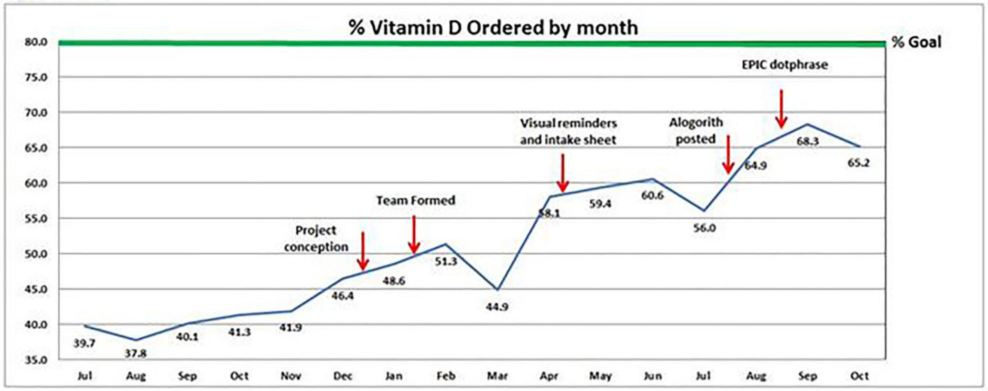

1. LeBlanc C, Ma J, et al, *Journal of Bone and Mineral Research.* 2015 2. Vojinovic J, Cimaz R, *Pediatric Rheumatology.* 2015
