# Supplementary material for: Improving Vitamin D Screening in a Pediatric Rheumatology Clinic Using Structured Quality Improvement Process
Source: Pediatr Qual Saf. 2022 Sep 8;7(5):e594. doi: 10.1097/pq9.0000000000000594 (PMC10997281; doi:10.1097/pq9.0000000000000594)
Supplement: Supplementary file 2 [file pqs-7-e594-s002.pdf]

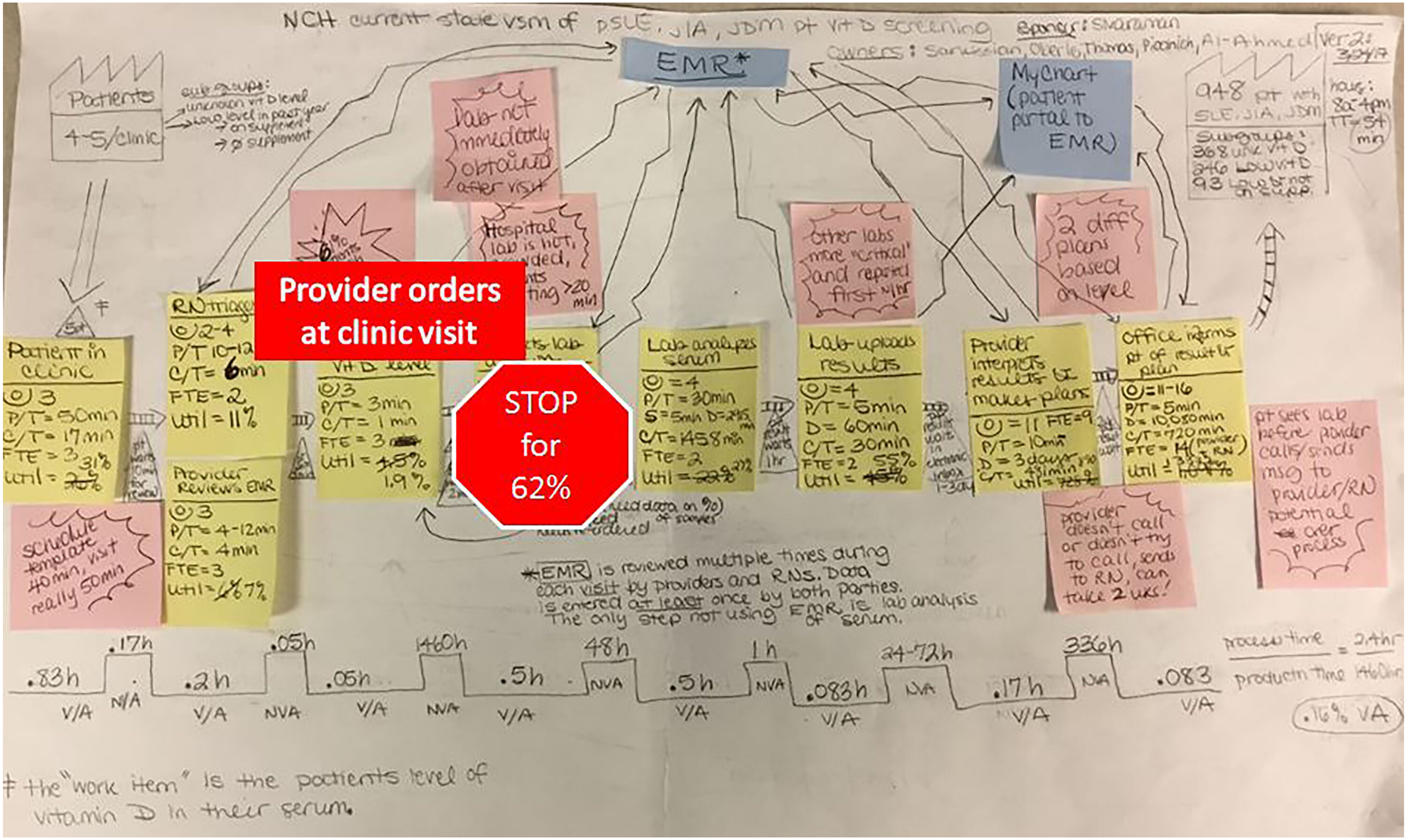
NCH current state VSM of pSLE, JIA, JDM pt Vit D screening
EMR*
MyChart (patient portal to EMR)
Patients
4-5/clinic
Patient in clinic
Provider orders
at clinic visit
STOP
for
62%
Lab analyzes serum
Lab uploads results
Provider interprets results & makes plans
Office informs pt of result & plan
948 pt with SLE, JIA, JDM
2 diff plans based on level
Other labs more "critical" and reported first
Hospital lab is hot
Lab not immediately obtained after visit
schedule template 40min, visit really 50min
Provider Reviews EMR
*EMR is reviewed multiple times during each visit by providers and RNs. Data is entered at least once by both parties. The only step not using EMR is lab analysis of serum.
process time = 2.4hr
.16% VA
the "work item" is the patients level of vitamin D in their serum.
