# Supplementary material for: Improving Vitamin D Screening in a Pediatric Rheumatology Clinic Using Structured Quality Improvement Process
Source: Pediatr Qual Saf. 2022 Sep 8;7(5):e594. doi: 10.1097/pq9.0000000000000594 (PMC10997281; doi:10.1097/pq9.0000000000000594)
Supplement: Supplementary file 3 [file pqs-7-e594-s003.pdf]

Figure 3. Nursing intake sheet

*Affix patient label*

Exam Room #

Temp

Pulse

BP

Weight

Height

Pain Score

Refill(s) needed?

Vaccine needed? (Flu, PCV13, PPSV23)

Licensed provider questions Y/N?

Eye care provider verified Y/N?

Vitamin D level within the year Y/N?

Result:

Notes
