# Supplementary material for: Improving Vitamin D Screening in a Pediatric Rheumatology Clinic Using Structured Quality Improvement Process
Source: Pediatr Qual Saf. 2022 Sep 8;7(5):e594. doi: 10.1097/pq9.0000000000000594 (PMC10997281; doi:10.1097/pq9.0000000000000594)

Supplementary Figure 5. Key driver diagram

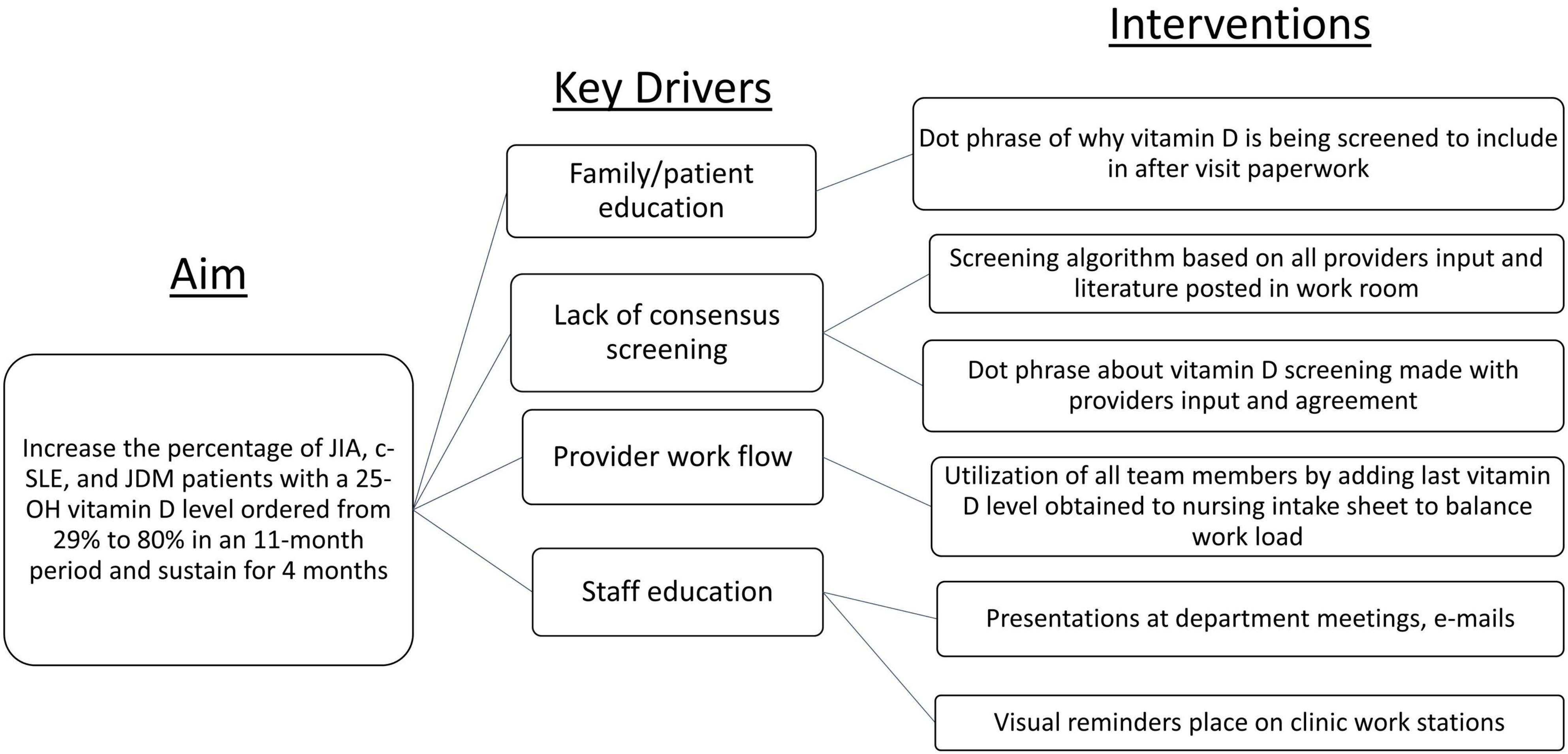

Supplement: Supplementary file 4 [file pqs-7-e594-s004.pdf]
